# MSX2 in pancreatic tumor development and its clinical application for the diagnosis of pancreatic ductal adenocarcinoma

**DOI:** 10.3389/fphys.2012.00430

**Published:** 2012-11-14

**Authors:** Kennichi Satoh, Shin Hamada, Tooru Shimosegawa

**Affiliations:** ^1^Division of Cancer Stem Cell, Miyagi Cancer Center Research InstituteNatori, Miyagi, Japan; ^2^Division of Gastroenterology, Tohoku University Graduate School of MedicineSendai, Miyagi, Japan

**Keywords:** pancreatic ductal adenocarcinoma, intraductal papillary-mucinous neoplasm of the pancreas, cancer development, MSX2, homeobox gene

## Abstract

MSX2, a member of the homeobox genes family, is demonstrated to be the downstream target for *ras* signaling pathway and is expressed in a variety of carcinoma cells, suggesting its relevance to the development of ductal pancreatic tumors since pancreatic ductal adenocarcinoma (PDAC) and intraductal papillary-mucinous neoplasia (IPMN) harbor frequent K-*ras* gene mutations. Recent studies revealed the roles of MSX2 in the development of carcinoma of various origins including pancreas. Among gastrointestinal tumors, PDAC is one of the most malignant. PDAC progresses rapidly to develop metastatic lesions, frequently by the time of diagnosis, and these tumors are usually resistant to conventional chemotherapy and radiation therapy. The molecular mechanisms regulating the aggressive behavior of PDAC still remain to be clarified. On the other hand, IPMN of the pancreas is distinct from PDAC because of its intraductal growth in the main pancreatic duct or secondary branches with rare invasion and metastasis to distant organs. However, recent evidence indicated that once IPMN showed stromal invasion, it progresses like PDAC. Therefore, it is important to determin how IPMN progresses to malignant phenotype. In this review, we focus on the involvement of MSX2 in the enhancement of malignant behavior in PDAC and IPMN, and further highlight the clinical approach to differentiate PDAC from chronic pancreatitis by evaluating MSX2 expression level.

## Introduction

Homeobox-containing genes regulate the morphological development of a variety of organs and their expression levels vary according to the development stages of organ (Wolgemuth et al., 1989; Morgan et al., 1992). The expression of MSX2, a member of the homeobox gene (Hox gene) family, is observed in a variety of sites, including premigratory cranial neural crest, tooth, and mammary gland, etc (Takahashi and Le Douarin, 1990; Davidson et al., 1991; Monaghan et al., 1991; Jowett et al., 1993; Davidson, 1995; Friedmann and Daniel, 1996; Phippard et al., 1996). The expression pattern of this gene in the development of organs suggests its pivotal role in epithelial–mesenchymal interactions (Satoh et al., 2004). On the other hand, accumulating evidence has revealed the active involvement of this gene in tumorigenesis and/or tumor development. MSX2 has been suggested to be a downstream target of the *Ras* signaling pathway because MSX2 was up-regulated in v-Ki-*ras* transfected NIH3T3 cells and antisense MSX2 cDNA and truncated MSX2 cDNA interfered with the transforming activities of both the v-K-*ras* and v-*raf* oncogene (Takahashi et al., 1996). In addition, the enhanced expression of MSX2 has been shown in a variety of carcinoma cell lines of epithelial origin compared to their corresponding normal tissues (Suzuki et al., 1993). In gastric cancer, MSX2 was identified as a cancer-specific hedgehog target and the down-regulation of this gene resulted in the inhibition of cancer cell growth *in vitro* (Ohta et al., 2009). Similarly, MSX2 has been shown to be a downstream target of WNT signal and has been correlated with the invasiveness of endometrioid adenocarcinoma (Zhai et al., 2011).

Pancreatic ductal adenocarcinoma (PDAC) is one of the most malignant gastrointestinal tumors. Once PDAC is clinically evident, it progresses rapidly to develop metastatic lesions, frequently by the time of diagnosis. Moreover, this carcinoma usually shows resistance to conventional chemotherapy and radiation therapy. Although recent molecular analyses of precursor lesions revealed an association between gene alterations and carcinogenesis (Hong et al., 2011), the pathogenic mechanisms that regulate the aggressive behavior of this cancer still remain to be clarified. On the other hand, intraductal papillary-mucinous neoplasia (IPMN) of the pancreas is a unique neoplasm that is considered to be a precancerous lesion analogous to adenomatous polyps of the colon (Loftus et al., 1996). IPMNs are classified as main duct type (MD-IPMN) or branch duct type (BD-IPMN) based on the location of the main tumor, detected by imaging studies or histology. BD-IPMN is likely to have a less aggressive biological behavior than MD-IPMN (Kobari et al., 1999; Terris et al., 2000; Doi et al., 2002; Hara et al., 2002; Kitagawa et al., 2003; Matsumoto et al., 2003; Sugiyama et al., 2003). However, the activation of oncogenes such as K-*ras* (Satoh et al., 1996), sonic hedgehog (Satoh et al., 2008b), and c-*erb* B-2 (Satoh et al., 1993a), accumulation of *p53* (Satoh et al., 1996), or the expression of a member of inhibitor of apoptosis family, survivin, (Satoh et al., 2001) as well as loss of chromosome 18q (Fukushige et al., 1998) in BD-IPMN, indicate the malignant potential of this neoplasm. Furthermore, stromal infiltration and distal metastasis have been reported even in this type of tumor (Sugiyama et al., 1998; Yasuda et al., 1998).

Until recently there has been little information about the expression or function of MSX2 in pancreatic tumors, although both PDAC and IPMN harbor frequent K-*ras* gene mutations at codon 12 (Satoh et al., 1993b, 1996) and MSX2 was suggested to be a downstream target of the *ras* signal. In this review, we summarize the recently identified roles and functions of MSX2 in the development of pancreatic tumors (PDAC and IPMN). We also demonstrate the validity of measuring the MSX2 expression level in clinical samples for the diagnosis of PDAC.

## Expression of MSX2 in pancreatic tissue

MSX2 was shown to be expressed in the expanding epithelia of the fetal murine pancreas, where PDX-1 was also detected, but not in the duct of the adult murine pancreas, suggesting that MSX2 might play a role in regulating the pancreatic developmental program (Kritzik et al., 1999). In cultured human cell lines, MSX2 expression was found in pancreatic cancer cell lines while it was not observed in human normal pancreatic duct epithelial cell line (HPDE) nor in pancreatic stellate cells (PSC) (Table 1) (Satoh et al., 2008a, 2010, 2011). Reverse transcription-polymerase chain reaction (RT-PCR) analysis of microdissected lesions also revealed that MSX2 expression occurred only in tumor lesions including carcinoma cells in PDAC tissues, and borderline to carcinoma cells in IPMN tissues, while no and faint expression of MSX2 transcripts was found in normal duct and adenoma cells of IPMN, respectively. The dominant MSX2 expression in cancer cells was also reported in other carcinomas such as bile duct (Ito et al., 2011), stomach (Ohta et al., 2009), and breast (Di Bari et al., 2009). Consistent with these findings, MSX2 expression is likely to be restricted to neoplastic duct cells in adult human pancreas.

**Table 1:** **Relative expression of MSX2 in cultured pancreatic cells (Satoh et al., 2008a, 2010)**.

**Cell**	**Relative MSX2 expression**
Panc-1	1
AsPC-1	0.87
MIAPaca2	0.3
BxPC3	0.02
Pancreatic stellate cell	0.01
Human pancreatic epithelial cell (HPDE)	<0.001

The correlation between the up-regulation of MSX2 and clinicopathological factors was investigated in a number of carcinomas. The expression of this gene was associated with good prognosis in breast carcinoma (Lanigan et al., 2010) and malignant melanoma (Gremel et al., 2011), while this gene expression was increased significantly in tumors with metastasis compared to those without metastasis in prostate carcinoma (Chua et al., 2010). In human PDAC tissues, frequent MSX2 expression in cancer cells was observed by immunohistochemistry and a significant correlation was found between MSX2 expression and histological differentiation and vascular invasion, whereas there was no association between this gene expression and the tumor stage (Table 2) (Satoh et al., 2008a), suggesting that MSX2 expression may be associated with the aggressiveness of PDAC because poor differentiation of PDAC is correlated with reduced survival time (Cleary et al., 2004).

**Table 2:** **Correlation between clinicopathologic findings and MSX2 expression (Satoh et al., 2008a)**.

	**MSX2 <30%**	**≥30%**	*P*-value^*^
Stage			0.957
I	1	2	
II	1	1	
III	4	4	
IV	8	11	
Histological classification			0.004
Well differentiated	8	2	
Moderately differentiated	6	9	
Poorly differentiated	0	7	
Vascular invasion			<0.0001
v0	0	3	
v1	6	1	
v2	8	1	
v3	0	12	

* Chi-square analysis.

## The effect of MSX2 on growth of pancreatic carcinoma and normal epithelial duct cells

It has been suggested that MSX2 may induce the proliferation of osteoprogenitors (Dodig et al., 1999), as well as osteoblasts (Liu et al., 1999), and this was gene also related to the enhancement of branching morphogenesis in mouse mammary ducts (Satoh et al., 2007). In addition, knockdown of MSX2 by small interfering RNA (siRNA) or small hairpin RNA (shRNA) inhibited the cell growth of gastric (Ohta et al., 2009) and ovarian (Zhai et al., 2011) carcinoma, indicating that the function of MSX2 is likely to be relevant to the regulation of the proliferation of epithelial cells as well as osteogenic cells. The involvement of MSX2 in normal pancreatic duct cell growth was examined using HPDE cells retrovirally transfected with MSX2 (Satoh et al., 2010). The effect of MSX2 on cell growth was analyzed by cell count every 48 h after seeding the cells. HPDE cells transfected with MSX2 demonstrated approximately 2.5-fold more cells compared to HPDE control cells at 4 days after seeding these cells. Similarly, forced expression of MSX2 in BxPC3 resulted in a significant induction of proliferation after 72 h of culture compared to control cells as determined by 5-bromo-2-deoxyuridine (BrdU) assay (Satoh et al., 2008a). In addition, MSX2 down-regulated Panc-1 cells by shRNA transfection significantly reduced the cellular growth rate. These findings clearly indicate that MSX2 facilitate the cellular growth of both benign and malignant pancreatic duct cells.

## MSX2 and invasion or metastasis of PDAC

Bone morphogenetic protein 4 (BMP4) has been shown to induce epithelial to mesenchymal transition (EMT) in PDAC cells and that MSX2 is indispensable for this phenomenon (Hamada et al., 2007). This raised the question of whether MSX2 itself could cause the EMT of PDAC cells. The involvement of this gene in the EMT of PDAC was investigated using MSX2 up- and down-regulated PDAC cells in gain and loss of function manners, respectively. As endogenous MSX2 expression was low in BxPC3 and high in Panc-1 cells (Table 1), several clones of BxPC3 stably overexpressing MSX2 and Panc-1 stably expressing MSX2 shRNA were generated (Satoh et al., 2008a). A significant morphological difference was observed between MSX2-transfected and control cells (parental BxPC3 and empty vector transfected cells). MSX2-expressing cells showed loose cell junctions, scattered morphology, and a more fibroblast-like appearance compared to control cells. A similar morphological change was observed between MSX2 expressing and down-regulated Panc-1 cells. MSX2 expressing parental Panc-1 and empty-vector transfected cells showed loose cell junctions and scattered morphology, while the MSX2 down-regulated cell lines demonstrated a cobblestone-like phenotype. By immunofluorescent staining, BxPC3 cells transfected with MSX2 exhibited a weakly diffuse distribution of E-cadherin and β-catenin in the cytoplasm, while control cells showed dominant membrane-bound staining. These molecular changes in MSX2 expressing cells are consistent with EMT. Consistent with the morphological and molecular changes, MSX2-expressing pancreatic cancer cells showed enhanced cell migration by wound healing scratch assay and two-chamber assay, while down-regulation of MSX2 in Panc-1 is associated with the suppression of cell migration. This evidence clearly indicates that MSX2 itself plays a crucial role in the EMT of PDAC cells (Satoh et al., 2008a). The effect of MSX2 on EMT was also investigated in mammary and ovarian cells. MSX2 transfected NMuMG cell, a spontaneously immortalized normal mouse mammary epithelial cell line, showed morphological and molecular changes consistent with EMT (Di Bari et al., 2009). MSX2-expressing NMuMG cells appeared spindle-shaped or fibroblast-like in the monolayer culture and showed reduced expression of the epithelial marker E-cadherin concomitant with the increased expression of mesenchymal markers vimentin and N-cadherin. In addition, forced expression of MSX2 in NMuMG cells resulted in the promotion of invasiveness. On the other hand, Zhai et al. demonstrated that ectopic expression of MSX2 also enhanced the invasiveness of ovarian carcinoma cells *in vivo* (Zhai et al., 2011). Since the expression of MSX2 in selected ovarian carcinoma cells induced changes suggestive of EMT but MSX2 expression was not consistently correlated with EMT markers in primary tumor specimens, they speculated that the involvement of MSX2 in EMT was complex and context-dependent. Therefore, although the involvement of MSX2 in tumor invasion was consistently observed in several kinds of carcinoma, the role of MSX2 in EMT might depend on the tumor species.

MSX2 expression also promoted cell migration or metastasis formation in an orthotopic environment. Control cells, MSX2 expressing cells and shMSX2 cells were injected into the pancreas of nude mice. Tumors were observed in the pancreas of mice implanted with all MSX2-expressing or shRNA-transfected MSX2 cells and control cells. MSX2-expressing cells frequently showed metastases to the liver and peritoneal dissemination, while control cells demonstrated no liver metastasis or only one peritoneal invasion (Table 3). By contrast, the metastases to the liver and peritoneal dissemination were suppressed in the mice injected with MSX2-inactivated cells (Table 3). In this context, MSX2 is likely to facilitate PDAC metastasis through the induction of EMT.

**Table 3:** **Summary of orthotopic implantation of MSX2-expressing or down-regulated cells in nude mice (Satoh et al., 2008a)**.

	**Metastasis to liver**	**Peritoneal dissemination**
BxPC3 control^#^ (*n* = 5)	0	1
MSX2 expressing BxPC3 (*n* = 5)	3^*^	5^*^
Panc-1 control^#^ (*n* = 5)	4^*^	3^*^
MSX2 down-regulated Panc-1 (*n* = 5)	0	0

# Control cells were transfected with empty vector;

* P < 0.05 (Chi-square analysis).

The mechanisms underlying the induction of EMT by MSX2 in PDAC cells were assessed by cDNA microarray, which identified the differentially expressed genes between control and MSX2 expressing cells (National Center for Biotechnology Information Gene Expression Omnibus database, GSE6585). Among the genes significantly up-regulated by MSX2, Twist1 was one of the most strongly induced genes in MSX2-expressing cells compared to control cells. Twist 1 was initially identified as a crucial regulator of embryonic morphogenesis in *Drosophilia* (Yang et al., 2004). A recent study revealed that Twist 1 is involved in invasion and/or metastasis through the induction of EMT in various types of carcinoma cells (Mironchik et al., 2005). In PDAC, immunohistochemical analysis showed that Twist 1 expression was correlated with MSX2 expression and the colocalization of these proteins was confirmed by double staining of fluorescence immunohistochemistry. In addition, nuclear expression of Twist 1 disappeared when MSX2 was down-regulated in Panc-1 cells (Satoh et al., 2008a). These findings suggest that MSX2 is likely to function in leading the PDAC cells to the state of EMT through the up-regulation of Twist 1.

## The role of MSX2 in the development of IPMN

IPMN is distinct from PDAC because it grows slowly and is rarely invasive, resulting in a better prognosis compared to PDAC (Loftus et al., 1996). However, recent evidence has indicated that once IPMN demonstrates stromal invasion, it progresses like PDAC (Sugiyama et al., 1998; Yasuda et al., 1998). Therefore, it is important to know how IPMN attains malignant phenotype. MSX2 is expressed in PDAC and its expression enhanced the aggressiveness of PDAC cells through the induction of EMT, as described above, and this gene is suggested to be a downstream target for *ras* signal (Takahashi et al., 1996). Since IPMN, like PDAC, harbors frequent K-*ras* mutations (Satoh et al., 1993b, 1996), the association of MSX2 expression with the tumor grade or clinicopathological features was examined in IPMN tissues to determine whether this gene could be involved in the process of benign-to-malignant progression in IPMN (Satoh et al., 2010). The expression levels of MSX2 mRNA in microdissected lesions from IPMNs were investigated by one-step quantitative real-time RT-PCR (QRT-PCR). The expression levels of MSX2 mRNA were increased in a stepwise manner from benign to malignant IPMN. Carcinoma lesions of IPMN expressed significantly higher levels of MSX2 mRNA than adenoma and borderline of IPMN cells did, while no significant difference was found between non-tumor lesions and adenoma-borderline IPMN cells. Consistent with the results of QRT-PCR, the immunoreactivity of MSX2 was frequently found in borderline IPMN (3/5, 60%), carcinoma of IPMN (12/19, 63.2%), and invasive carcinoma derived from IPMN (5/5, 100%), while its expression was seen in only one of 16 adenoma of IPMN tissues. When multivariate analysis among seven clinical parameters, including age, sex, branch duct size, nodule size, diameter of main pancreatic duct, serum CEA and CA19-9 levels, in addition to MSX2 expression, was done, MSX2 expression was identified as the only independent factor that predicted malignant BD-IPMN (Table 4).

**Table 4:** **Predictive factors for malignant IPMN by multivariate analysis (Satoh et al., 2010)**.

**Parameter**	**Adjusted odds ratio (confidence interval)**
Age (≥70)	0.38 (0.06 – 2.35)
Sex	0.51 (0.08 – 3.28)
Branch (≥30 mm)	2.90 (0.46 – 18.26)
Main pancreatic duct (≥6 mm)	2.01 (0.24 – 16.65)
Nodule (≥6 mm)	2.99 (0.39 – 22.65)
CEA (>5)	0.65 (0.08 – 5.22)
CA19-9 (>37)	6.91 (0.12 – 394.4)
MSX2 expression	8.19 (1.4 – 47.9)^*^

* P < 0.02 (Log rank regression analysis).

A branch duct size cutoff of 30 mm has been widely accepted as a factor for predicting the malignancy of BD-IPMN (Tanaka et al., 2006). However, its low sensitivity for malignancy has also been reported (Pelaez-Luna et al., 2007). Thus, more specific predictive factors for malignant BD-IPMN were explored by various approaches. Clinical findings such as branch size, presence of nodule or dilatation of the main pancreatic duct have been described as signs of malignant BD-IPMN (Kitagawa et al., 2003; Sugiyama et al., 2003; Kobayashi et al., 2005; Pelaez-Luna et al., 2007). Similarly, molecular events including mutation of K-*ras* (Satoh et al., 1993b, 1996), inactivation of *p53* (Satoh et al., 1996) or *smad4* (Biankin et al., 2002) were demonstrated to be correlated with malignant BD-IPMN. However, it is known whether molecular markers would be better predictive factors for malignant BD-IPMN than clinical parameters such as nodule size or dilatation of a branch duct. Based on the above findings, MSX2 expression was found to be a better predictive factor for carcinoma of IPMN compared to the clinical parameters that were previously reported to be relevant to malignant IPMN.

## MSX2 and chemoresistance of PDAC

Carcinoma tissues are known to consist of a heterogeneous cellular population containing a minor population of permanent proliferating cells and a major population of differentiated cells with limited proliferation potential. Among the permanent proliferating cells, so-called cancer stem cells (CSCs) are considered to be responsible for the initiation, metastasis, chemoresistance and recurrence of tumor (Reya et al., 2001). Recently, the induction of a breast CSC phenotype by the forced expression of Snail and Twist, which leads to EMT, has been demonstrated (Mani et al., 2008). Since increased expression of MSX2 induced EMT and enhanced the metastasis of PDAC cells, MSX2 is likely to have pivotal role in maintaining the characteristics of CSCs. Thus, the involvement of MSX2 in chemoresistance, which is one of the characteristics of CSCs in PDAC, was investigated (Hamada et al., 2012). To assess the association between MSX2 and chemoresistance, MSX2-expressing and down-regulated PDAC cells were treated with gemcitabine or 5-FU. The survival of MSX2 expressing PDAC cells was approximately twofold greater than that of control cells, while MSX2 down-regulated cells showed 30–50% decreases in cell viabilities after gemcitabine or 5-FU treatment. Furthermore, forced or reduced expression of MSX2 in PDAC gave rise to increased or decreased numbers of side population (SP) cells, which have been shown to be associated with CSCs (Dean et al., 2005; Golebiewska et al., 2011), respectively. Interestingly, the chemoresistance of PDAC cells by MSX2 expression was abolished when ATP-binding cassette transporter ABCG2, identified as one of the MSX2 target genes by cDNA microarray as described above, was down-regulated by siRNA transfection. Based on these findings, it is suggested that MSX2 enhances the chemoresistance through ABCG2 induction and the increase in the CSC phenotype.

## Clinical application for diagnosis of PDAC by measuring MSX2 expression level

Endoscopic pancreatic duct brushing is a convenient diagnostic method for strictures in the main pancreatic duct or in the second branch. However, the diagnostic sensitivity of this method for PDAC is shown to be low (40–70%) (McGuire et al., 1996; Vandervoort et al., 1999; Pugliese et al., 2001; Uchida et al., 2007). Since MSX2 expression is limited to neoplastic duct cells in the adult pancreas, the detection of MSX2 could be a useful marker for the diagnosis of PDAC. Therefore, the expression level of MSX2 mRNA was investigated in 95 endoscopic brushing samples from stricture of the pancreatic duct to determine whether MSX2 expression could distinguish malignant from benign pancreatic diseases and improve the diagnostic yield of brush cytology (Satoh et al., 2011). The samples were collected when ductal strictures were found during ERCP using cytology brushes with 0.025–0.035 inch guide wire. QRT-PCR was carried out on each sample by adding the same amount of total RNA.

In 13 of 95 patients (13.7%), cytological brushing could not be done because the guide wire could not be passed through the ductal stricture. In the remaining 82 patients, endoscopic brushing was successfully carried out and satisfactory specimens were obtained from all cases. Final diagnoses were PDAC (*n* = 57) and chronic pancreatitis (CP, *n* = 25). The sensitivity of routine brush cytology for PDAC was 47.4% (27/57) with 100% specificity and 63.4% diagnostic accuracy (Table 5). MSX2 mRNAs in brushing samples were successfully detected and quantified by normalization to the respective glyceraldehyde-3-phosphate dehydrogenase (GAPDH) copy number. The mean expression level of MSX2 mRNA was significant higher in PDAC samples than benign strictures (Table 6). The MSX2 expression level was judged positive when it was equal to or higher than the cut-off value which was defined by the receiver operating characteristic curve. Using this cut-off value, the sensitivity, specificity for malignancy, and accuracy for diagnosis were 73.7, 84.0, and 79.3%, respectively (Table 5). The diagnostic sensitivity for PDAC and the accuracy by analyzing the MSX2 expression levels were much higher than those by cytological examination. The diagnostic sensitivity or accuracy by the evaluation of MSX2 expression compared favorably to other markers such as telomerase (Ohuchida et al., 2005) or MUC1 (Wang et al., 2007) and is similar to the combination analysis of the DNA concentration of methylated cyclin D2, NPTX2, and TFPI2 promoter in brush cytological samples (Parsi et al., 2008). Although the K-*ras* mutation was reported to be more frequently found in brush samples (Van Laethem et al., 1995; Pugliese et al., 2001), it is difficult to use the the K-*ras* mutation as a tool to differentiate PDAC from CP since this mutation is also frequently detected in CP (Yanagisawa et al., 1993). For example, the K-*ras* mutation was frequently detected in both cancer (87%) and pancreatitis (40%) when brush samples from 34 cases of PDAC and 11 of CP were analyzed (Pugliese et al., 2001). In this context, the evaluation of the MSX2 expression level could be a useful tool for differentiating PDAC from CP when a stricture is found in the pancreatic duct.

**Table 5:** **Comparison between cytology and MSX2 measurement in brush samples (Satoh et al., 2011)**.

	**Sensitivity (%)**	**Specificity (%)**	**Accuracy (%)**
Cytology	47.4	100	63.4
MSX2 evaluation	73.7	84.0	79.3

**Table 6:** **MSX2 expression levels in ERCP brush samples (Satoh et al., 2011)**.

	**Number of samples**	Mean MSX2 expression level^*^	***P*-value^#^**
PDAC	57	0.012, 0.0024	<0.0001
CP	25	0.0026, 0.0004	−

* MSX2/GAPDH (copy number/μl, mean ± standard error).

# Mann–Whitney U-test.

## Conclusion

**Figure 1:**
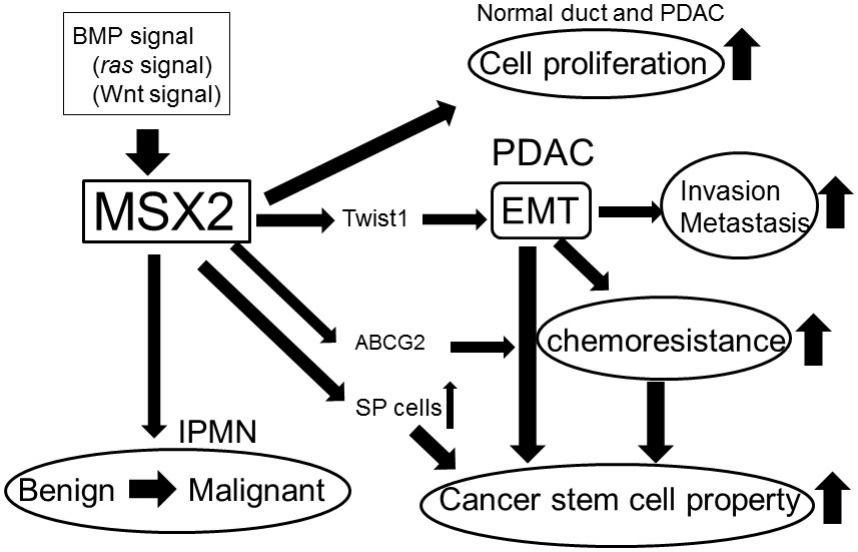
**Summary of the roles of MSX2 in pancreatic tumor development**.

Recent studies have clarified the functions of MSX2 in pancreatic tumor development. MSX2 appears to enhance the malignant phenotype of PDAC by stimulating cell proliferation, the induction of EMT and an increase in the characteristics of CSCs (Figure 1). MSX2 also plays an important role in enhancing the aggressiveness of BD-IPMN, indicating that this gene may be a good therapeutic target in pancreatic tumors. Moreover, measurement of the MSX2 expression level in endoscopic brush samples enabled the differentiation of malignant strictures in the pancreatic duct from benign ones, suggesting that the evaluation of MSX2 could be applied clinically for the diagnosis of malignant pancreatic tumor.

### Conflict of interest statement

The authors declare that the research was conducted in the absence of any commercial or financial relationships that could be construed as a potential conflict of interest.

## Abbreviations

PDAC: pancreatic ductal adenocarcinoma
IPMN: intraductal papillary-mucinous neoplasia
MD-IPMN: main duct type intraductal papillary-mucinous neoplasia
BD-IPMN: branch duct type intraductal papillary-mucinous neoplasia
HPDE: human normal pancreatic duct epithelial cell line
PSC: pancreatic stellate cells
RT-PCR: reverse transcription-polymerase chain reaction
siRNA: small interfering RNA
shRNA: small hairpin RNA
BrdU: 5-bromo-2-deoxyuridine
BMP4: Bone morphogenetic protein 4
EMT: epithelial to mesenchymal transition
QRT-PCR: quantitative real-time reverse transcription-polymerase chain reaction
CSC: cancer stem cell
SP: side population
CP: chronic pancreatitis
GAPDH: glyceraldehyde-3-phosphate dehydrogenase.
